# Supplementary material for: Towards unified AI-driven fracture mechanics: the extended deep energy method (XDEM)
Source: Nat Commun. 2026 Aug 15;17:8492. doi: 10.1038/s41467-026-76748-1 (PMC13484471; doi:10.1038/s41467-026-76748-1)
Supplement: Supplementary file 3 [file 41467_2026_76748_MOESM3_ESM.zip › pic/Appendix/1D_crack/1D_crack.pdf]

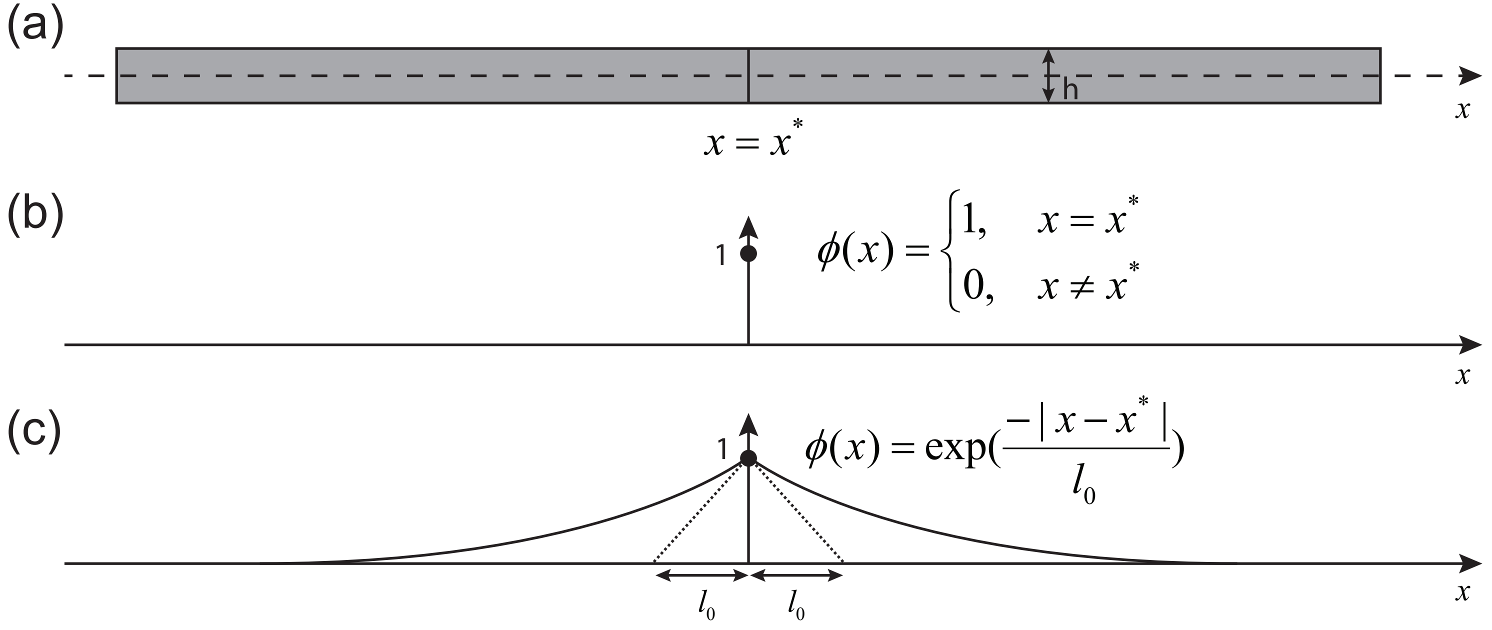
(a)
h
x
$x = x^*$
(b)
1
$\phi(x) = \begin{cases} 1, & x = x^* \\ 0, & x \neq x^* \end{cases}$
x
(c)
1
$\phi(x) = \exp(\frac{-|x - x^*|}{l_0})$
l_0
l_0
x
